# Tools to help healthcare professionals recognize palliative care needs in patients with advanced heart failure: A systematic review

**DOI:** 10.1177/0269216320963941

**Published:** 2020-10-15

**Authors:** Stephanie MC Ament, Inge ME Couwenberg, Josiane JJ Boyne, Jos Kleijnen, Henri EJH Stoffers, Marieke HJ van den Beuken, Yvonne Engels, Louise Bellersen, Daisy JA Janssen

**Affiliations:** 1 Department of Health Services Research, Care and Public Health Research Institute, Maastricht University, Maastricht, The Netherlands; 2 Department of Cardiology, Catharina Hospital, Eindhoven, North Brabant, The Netherlands; 3 Department of Patient and Care, Maastricht University Medical Centre, Maastricht, Limburg, The Netherlands; 4 Department of Family Medicine, Care and Public Health Research Institute, Maastricht University, Maastricht, The Netherlands; 5 Kleijnen Systematic Reviews Ltd, York, UK; 6 Centre of Expertise for Palliative Care, Maastricht University Medical Centre, Maastricht, The Netherlands; 7 Department of Anesthesiology, Pain and Palliative Medicine Radboud University Medical Centre, Nijmegen, The Netherlands; 8 Department of Cardiology, Radboud University Medical Centre, Nijmegen, The Netherlands; 9 Department of Research and Education, Ciro, Horn, The Netherlands

**Keywords:** Systematic review, palliative care needs, heart failure, assessment, screening tool, advanced stage, end of life care, knowledge translation

## Abstract

**Background:**

The delivery of palliative care interventions is not widely integrated in chronic heart failure care as the recognition of palliative care needs is perceived as difficult. Tools may facilitate healthcare professionals to identify patients with palliative care needs in advanced chronic heart failure.

**Aim:**

To identify tools to help healthcare professionals recognize palliative care needs in patients with advanced chronic heart failure.

**Design:**

This systematic review was registered in the PROSPERO database (CRD42019131896). Evidence of tools’ development, evaluation, feasibility, and implementation was sought and described.

**Data sources:**

Electronic searches to identify references of tools published until June 2019 were conducted in MEDLINE, CINAHL, and EMBASE. Hand-searching of references and citations was undertaken. Based on the identified tools, a second electronic search until September 2019 was performed to check whether all evidence about these tools in the context of chronic heart failure was included.

**Results:**

Nineteen studies described a total of seven tools. The tools varied in purpose, intended user and properties. The tools have been validated to a limited extent in the context of chronic heart failure and palliative care. Different health care professionals applied the tools in various settings at different moments of the care process. Guidance and instruction about how to apply the tool revealed to be relevant but may be not enough for uptake. Spiritual care needs were perceived as difficult to assess.

**Conclusion:**

Seven tools were identified which showed different and limited levels of validity in the context of palliative care and chronic heart failure.

**What is already known about the topic?**

- Identification of palliative care needs in patients with chronic heart failure may be more appropriate for the delivery of optimal care than the application of prognostic models to estimate the risk of dying.
- Interdisciplinary palliative care interventions in addition to regular heart failure care have a positive impact on quality of life, patient satisfaction, advance care planning, and cost-minimization.

**What this paper adds?**

- Seven tools were identified to help healthcare professionals to recognize palliative care needs in patients with chronic heart failure.
- The identified tools differ in purpose, content, and user.
- The validation of the tools and the validation research specifically for the context of chronic heart failure is limited.
- Guidance and education for using the tool are needed for implementation of a tool in the context of advanced chronic heart failure.

**Implication for practice, theory or policy**

- Validated tools are needed to help healthcare professionals to recognize palliative care needs in patients with chronic heart failure.
- Policy makers, guideline developers and quality improvement experts must be aware of the purpose and prior conditions of existing tools in the context of chronic heart failure before integrating them in policy, guidelines or local work appointments.

## Introduction

Chronic heart failure is a major contributor to global morbidity and mortality. It affects around 26 million people worldwide, and its prevalence increases due to new treatments, life style changes and an aging population.^1^ Despite the advances in chronic heart failure treatment, patients may live years with symptoms such as breathlessness, fatigue, tiredness, and poor appetite after diagnosis^2–4^ and have at least the same level of palliative care needs as patients with cancer.^5^ Research has shown that palliative care in the context of advanced chronic heart failure has a positive effect on patient-centered outcomes, documentation of care preferences, and resource use.^6^ Therefore, there is a need to integrate palliative care into advanced chronic heart failure care to reduce symptom burden and to improve quality of life.

Unfortunately until today, palliative care has not been routinely implemented in current heart failure practice.^7,8^ Healthcare professionals experience difficulties in recognizing palliative care needs in this patient group. Some research has focused on prognostication and the use of the Surprise Question to identify patients with advanced chronic heart failure that may be in need for palliative care.^9–11^ However, Janssen et al. described the role and the limited value of using prognostic tools in the context of recognition of palliative care needs.^12^ The need for palliative care must not be limited to a particular prognosis as patient needs differ and as the disease trajectory is difficult to predict.^9^ In addition, the European Association for Palliative Care recently recommended that palliative care must be available for all advanced chronic heart failure patients with palliative care needs, regardless of their prognosis.^13^

Given the current practice gap in recognizing palliative care needs in patients with advanced chronic heart failure, this systematic review aims to identify structured tools that can help healthcare professionals perform this task. Earlier reviews have been done to identify tools for timely recognition of palliative care needs. However, they were not systematically performed,^12^ or focused only on general practice^15^ or were not specifically focused on advanced chronic heart failure.^16^

Therefore, our specific objectives were: (1) to identify the available tools for healthcare professionals to identify palliative care needs in advanced chronic heart failure; (2) to describe the characteristics of the identified tools; (3) to describe the level of validity of the available tools regarding palliative care needs and advanced chronic heart failure; and (4) to describe the level of feasibility and the level of implementation of the tools including the lessons learned.

## Methods

This systematic review was performed using the recommendations in the Cochrane Handbook where applicable. The Preferred Reporting Items for Systematic Reviews and Meta-Analyses statement was used for reporting this systematic review (see Supplemental File 1).^17,18^ The protocol is registered with PROSPERO (CRD42019131896).

### Eligibility criteria

#### Types of tools

The tools being included are defined as a collection of questions, scales or other means of obtaining information which together provide guidance for the screening and identification of patients’ palliative care needs. Prognostic tools (including the Surprise Question) were excluded as they are intended to identify patients nearing end of life.^19,20^ Also, tools developed solely to measure health outcomes were excluded from this review.

#### Users of tools

Users are healthcare professionals caring for patients with chronic heart failure or patients with chronic heart failure. Exclusion: patients under 18 years of age.

#### Context

Any healthcare setting was included.

#### Types of studies

Studies describing the development, evaluation, and implementation of tools to enable identification of patients with chronic heart failure experiencing palliative care needs were included. Implementation studies had to describe strategies to promote the adoption and integration of tools into specific routine practices.^21^ There was no restriction on language. Study protocols, conceptualizations, debates, case reports, narrative reviews, and systematic reviews were excluded.

### Information sources

An electronic literature search was conducted utilizing PubMed, MEDLINE (OvidSP) (1946–June 2019), CINAHL (EBSCO Host) (1982–June 2019), and Embase (OvidSP) (1980–June 2019) to identify tools. Free text terms and MeSH terms regarding “tools,” “palliative care,” and “heart failure” were used (for search strategy, see Supplemental File 2). Reference lists of retrieved relevant reviews were screened for additional references. Also, databases such as PubMed and the Web of Science were used to screen for publications citing the included references. A second electronic search was performed as a methodological check to make sure we identified all evidence with respect to the tools identified and lessons learned in practice (September 2019). Free text terms and MeSH terms regarding the tools identified, and “implementation” were used (for search strategy, see Supplemental File 3). An information expert (J.K.) checked both electronic search strategies.

### Study selection

Two researchers screened study titles and abstracts independently (S.A. screened all, Y.E. and L.B. screened both half). Thereafter, each retrieved full text paper was screened by two researchers independently (S.A. screened all, and M.v.B., D.J., J.B. screened all one-third). Authors of conference abstracts received an email and were asked to send the full research paper. Any disagreements were resolved by discussion between two researchers and if necessary with a third researcher. The study selection of the second electronic search was performed by one researcher (S.A.).

### Data extraction

A data extraction form was developed. Two researchers (I.C. and S.A.) independently extracted the data and disagreements were resolved by discussion. Study characteristics, tool characteristics, the level of feasibility and implementation, and feasibility and implementation lessons learned were extracted. The described content, construct and criterion validity activities and results were extracted and analyzed based on the definitions of COnsensus-based Standards for the selection of health status Measurement InstrumeNts (COSMIN) (H.S. and S.A.).^22^ Content validity is the degree to which the content of an instrument is an adequate reflection of the construct to be measured. Construct validity is the degree to which the scores of an instrument are an adequate reflection of the dimensionality of the construct to be measured. Criterion validity is the degree to which the scores of an instrument are an adequate reflection of a gold standard. The methodology for the development and design of the tools, specifically focused on chronic heart failure, was assessed based on standard components for development and implementation of medical checklists (I.C. and S.A.).^23^ Grol et al.’s Characteristics of Innovations Framework was used as a topic list for the qualitative data-extraction and analyses of the practice-based factors that might promote or hinder the tool’s uptake in practice.^24^ A narrative synthesis was used to describe the findings.

## Results

**Figure 1.**
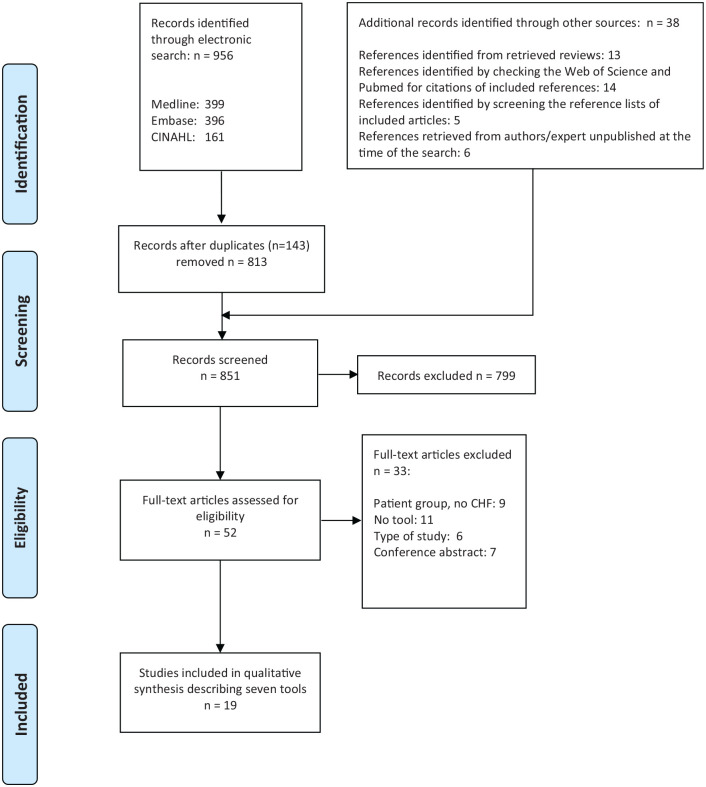
PRISMA flow diagram.

The flow chart of the search process for the included studies is provided in Figure 1. A total of 851 records was identified after the duplicates were removed. Thirteen records were identified by screening the reference lists of the retrieved systematic reviews.^13,15,16,25–27^ Based on a second electronic search to check whether we identified all evidence, no additional papers were included. The Supportive and Palliative Care Indicators Tool (SPICT) was identified in a conference abstract via the electronic search and was integrated in this second electronic search. As we did not receive more details about the conference reference, and as we did not find other references regarding the SPICT in chronic heart failure, we excluded this tool from this review.

### Research aim 1: Identified tools

A total of 19 papers were included (Table 1). These studies described seven tools: the POS/IPOS (Integrated Palliative care Outcome Scale),^28–30^ the Needs Assess-ment Tools Progressive Disease—Heart Failure (NAT: PD-HF),^31,32^ the RADboud indicators for PAlliative Care Needs (RADPAC),^33–38^ the Heart Failure Needs Assess-ment Questionnaire (HFNAQ),^39^ the Care related Quality of Life for Chronic Heart Failure Questionnaire (CareQol CHF),^40,41^ the Heart Failure Palliative Approach to Care (HeFPAC),^42,43^ and the Nececidades Paliativas (NECPAL).^44–46^

**Table 1.** Study characteristics.

Tool	Author	Country	Type of study
Integrated palliative care outcome scale (IPOS/POS)	Kane et al.^28^	Ireland	Feasibility
	Kane et al.^29^	Ireland	Feasibility
	Oriani et al.^30^	Ireland United kingdom	Evaluation
Needs assessment tools progressive disease—heart failure (NAT: PD-HF)	Waller et al.^31^	Australia	Development and feasibility
	Janssen et al.^32^	The Netherlands	Feasibility
RADboud indicators for PAlliative Care Needs (RADPAC)	Thoonsen et al.^38^	The Netherlands	Implementation
	Thoonsen et al.^33^	The Netherlands	Development
	Thoonsen et al.^36^	The Netherlands	Implementation
	Thoonsen 2016.^36^	The Netherlands	Implementation
	Thoonsen 2016.^37^	The Netherlands	Development and implementation
	Thoonsen et al.^34^	The Netherlands	Implementation
Heart failure needs assessment questionnaire (HFNAQ)	Davidson et al.^43^	Australia	Development
	Davidson et al.^39^	Australia	Evaluation
Care related quality of life for chronic heart failure questionnaire (CareQol CHF)	Van Kessel et al.^40^	The Netherlands	Development
	Van Kessel^41^	The Netherlands	Development
Heart failure palliative approach to care (HeFPAC)	Strachan et al.^42^	Canada	Development
Nececidades paliativas (NECPAL)	Gomez et al.^44^	Spain	Development
	Orzechowski et al.^45^	Brazil	Evaluation
	Gastelurrutia et al.^46^	Spain	Evaluation

### Research aim 2: Characteristics and types of tools

The tools varied in purpose and in the content elements (Table 2). Four tools were identified as healthcare professionals-completed tools (NAT: PD-HF, RADPAC, NECPAL, and HeFPAC). The HeFPAC is specifically developed for nurses and RADPAC is specifically developed for general practitioners. The IPOS, CareQol CHF, and HeFNAQ were identified as patient-completed tools.

**Table 2.** Tool characteristics.

Tool	Aim of the tool	User	Type of tool	Screening/case finding	CHF specific needs	Palliative care dimensions (of domains)				
						Physical	Psychological	Social	Spiritual	Other
Integrated palliative care outcome scale (IPOS/POS)	To capture palliative symptoms and concerns	Patient	Patient reported outcome measurement	Screening	No	Yes	Yes	Yes	Yes	Information financial caregiver needs
Needs assessment tools progressive disease—heart failure (NAT: PD-HF)	To assist health professionals in matching the types and levels of need experienced by patients and their caregivers with the most appropriate person or service to address identiﬁed needs	Professional	Needs assessment tool	Screening	Yes	Yes	Yes	Yes	Yes	CultureFinancial/legal CaregiverInformationSpecialized palliative care
RADboud indicators for PAlliative Care Needs (RADPAC)	To help general practitioners in the identification of patients with CHF, COPD, or cancer, in need of palliative care	Professional	Set of indicators	Case finding	No	Yes	No	No	No	NA
Heart failure needs assessment questionnaire (HFNAQ)	To assess the needs of patients with CHF on four dimensions: physical, psychological, social, and existential, and to assist in their care planning and monitor the efficacy and impact of interventions	Patient	Patient reported outcome measurement	Screening	Yes	Yes	Yes	Yes	Yes	Information financial
Care related quality of life for chronic heart failure questionnaire (CareQol CHF)	To measure care-related quality of life in patients diagnosed with chronic heart failure	Patient	Patient reported outcome measurement	Screening	Yes	Yes	Yes	Yes	No	Feeling of being in safe hands
Heart failure palliative approach to care (HeFPAC)	To inform CHF-specific assessments and care	Professional	Checklist	Screening	Yes	Yes	No	No	No	Caregiver InformationHome care servicesAdvanced care planning
Nececidades Paliativas (NECPAL)	To identify persons in advanced-terminal disease state and requiring palliative care in health and social services	Professional	Set of indicators	Case finding and screening	No	Yes	Yes	No	No	Specialized palliative care

AkPs: Australia-modified karnofsky Performance scale; (FACQ-PC): Caregiving Questionnaire for Palliative Care; CHF: chronic heart failure; COPD: chronic obstructive pulmonary disease; ESAS: Edmonton Symptom Assessment System; GPs: general practitioners; NA: not applicable.

All tools included the physical domain, five tools included the psychological domain (IPOS, NAT: PD-HF, HFNAQ, CareQol CHF, NECPAL) and four tools included the social domain (IPOS, NAT: PD-HF, HFNAQ, CareQol CHF). Spirituality was only included in three tools (NAT: PD-HF, IPOS, HFNAQ). Four chronic heart failure specific tools were identified: NAT: PD-HF, HFNAQ, CareQol CHF, and the HeFPAC. The RADPAC, NECPAL, and HeFPAC have chronic heart failure specific clinical indicators for increased risk of possible palliative care needs. The NAT: PD-HF was developed to assist health professionals in matching the types and levels of needs experienced by people with advanced chronic heart failure and their caregivers with the most appropriate person or service to address identiﬁed needs. This tool was specifically developed to capture the patient’s and caregiver’s needs during one assessment.

The NAT: PD-HF, the CareQol CHF, the NECPAL, and the HeFPAC have an instruction on how to use the tools in practice as part of the tool itself. The NAT: PD-HF, the NECPAL, and the HeFPAC include also recommendations for the next actions to be taken such as consideration of specific patient information (HeFPAC), guiding to a “six steps for palliative care provision” (NECPAL) or referral to specialized palliative care (NAT:PD-HF). The HeFPAC includes a definition of palliative care and the design of the tool was a fundamental part of the development process.^42^

### Research aim 3: Development and validation

Different development process steps specifically for chronic heart failure were described. The HeFPAC was the only tool which was developed based on a needs assessment of the target group of the tool (nurses) and the chronic heart failure context.^42^ Before the development of this tool, nurses requested a “hands-on” practice tool that would increase their ability to care for heart failure patients with palliative care needs. The specific aim and future users were determined before the start of the development of the HeFPAC and the RADPAC.^33,37,42^

Content validity research in the context of chronic heart failure and palliative care was performed regarding one patient reported outcome measurement: the IPOS,^29,30^ and three tools that need to be completed by healthcare professionals: the NAT: PD-HF,^31^ the RADPAC,^33,35^ and the HeFPAC^42^ (Table 3). According to Kane et al., the general IPOS items reflected the patient’s chronic heart failure experience.^29^ Oriani et al. showed that 77% of the main problems in the open question of the IPOS were reflected in the closed questions.^30^ The authors suggested that adaptation and further psychometric validation is needed. In comparison with the oncological version, the NAT:PD-HF was adapted regarding medication and treatment regimens.^31^ No study described a final multidisciplinary review about the items in the context of chronic heart failure and palliative care.

**Table 3.** Level of tool validity for palliative care and chronic heart failure.

Tool	Validity assessment performed	
	Content	Criterion
Integrated palliative care outcome scale (IPOS/POS)	Yes. Kane et al.^29^: interviews Oriani et al.^30^: secondary analysis of three studies	No
Needs assessment tools progressive disease—heart failure (NAT: PD-HF)	Yes. Waller et al.:^31^ Multidisciplinary expert panel	Yes. Waller et al.^31^: levels of physical (*p* = 0.039), daily living (*p* = 0.001) and spiritual/existential (*p* = 0.038) concerns were correlated with the Heart failure needs assessment (HFNAQ) item scores; levels of psychological (*p* = 0.155) and social (*p* = 0.304) concerns not. Janssen et al.^32^: levels of physical (*p* = 0.12), psychological (*p* = 0.71), daily living (*p* = 0.38) and caregiver distress (*p* = 0.33) concerns were not correlated with respectively the ESAS summary score, the ESAS distress score, the AKPS score the FACQ-PC caregivers distress score
RADboud indicators for PAlliative care needs (RADPAC)	Yes. Thoonsen et al.^33^: Literature review, focus groups with general practitioners and experts in the field, rand delphi process Thoonsen et al.^35^: Interviews	No
Heart failure needs assessment questionnaire (HFNAQ)	No	No
Care related quality of life for chronic heart failure questionnaire (CareQol CHF)	No	No
Heart failure palliative approach to care (HeFPAC)	Yes. Strachan et al.^42^: literature review, focus groups and feedback	No
Nececidades paliativas (NECPAL)	No	No

AKPS: Australia-modified Karnofsky Performance scale; ESAS: Edmonton Symptom Assessment System; FACQ-PC: Family Appraisal of Caregiving Questionnaire for Palliative Care; HFNAQ: Heart Failure Needs Assessment.

Criterion validity in the context of chronic heart failure and palliative care was assessed for one tool, namely the NAT: PD-HF. Waller et al. used the HFNAQ as the gold standard and showed that the levels of physical, daily living, spiritual concern items were significantly correlated with the HFNAQ item scores.^31^ The levels of psychological and social concern items were not significantly correlated. Janssen et al. used the Edmonton Symptom Assessment System (ESAS), Australia-modified karnofsky Performance scale (AkPs), and the Caregiving Questionnaire for Palliative Care (FACQ-PC) as gold standards regarding the NAT: PD-HF level of concern items. Criterion validity was slightly shown for the physical item, but was not confirmed for the psychological, daily living, and caregiver distress items.

No paper analyzed the construct validity of the tools.

### Research aim 4: Feasibility and implementation

Twelve references focused on applying the tools in practice.^28,29,31,32,34–39,45,46^ One paper performed a secondary analysis of existing POS/IPOS data collected in three studies and combined data based on multidisciplinary use.^30^

#### Level of feasibility

The IPOS and the NAT: PD-HF are the only tools that have been tested to identify palliative care needs in heart failure practice.^28,29,31,32^ Acceptability and feasibility of the IPOS and the NAT-PD:HF among health care professionals were evaluated in four papers.^28,29,31,32^

As part of a preliminary evaluation of the NAT-PD:HF, Waller et al. piloted the NAT-PD:HF and concluded that the tool could be completed in a “reasonable time” within clinical practice. Janssen et al. showed that the NAT-PD:HF was not fully acceptable and feasible to Dutch heart failure nurses for timely recognition of palliative care needs, because time of completing the NAT-PD:HF (mean 26 SD 12 min) was perceived as too time consuming for assessment of palliative care needs as part of care as usual in their setting.^32^ According to their experiences with the NAT-PD:HF, heart failure nurses missed concrete questions to ask the items. The heart failure nurses highlighted the integration of the family caregiver needs as part of the NAT-PD:HF as a strength.

The IPOS showed to be acceptable and feasible to the nurses and patients to identify palliative symptoms and concerns. Kane et al. showed that the comprehensive list of symptoms, vocabulary prompts and open questions in the IPOS was perceived to facilitate patients to identify personal symptoms and needs that they had not identified themselves. The nurses acknowledged that their focus and actual priorities to assess chronic heart failure related physical symptoms differed from the IPOS questions and outcomes. The IPOS was perceived as a tool to facilitate the individual experience of advanced chronic heart failure and as a method to give voice to the patient during a consultation. Oriani et al. concluded that, the first open question of the IPOS/POS (“What have been your main problems or concerns over the past week?”) is valuable to identify unique and personal needs.

#### Feasibility: Setting and timing

The tools were applied in different healthcare settings and at different moments (Table 4). An optimal setting was mentioned as a facilitator to recognize and discuss palliative care needs.^32^ In four studies, the IPOS, the NAT-PD:HF or NECPAL were used during a face-to-face consultation.^28,29,31,32,46^ According to Kane et al., the IPOS could be very well integrated in an outpatient consultation at a busy clinic.^28,29^ Heart failure nurses who tested the NAT-PD:HF mentioned that a home visit was seen as an opportunity to discuss palliative care needs and that a telephone consult would not be appropriate for recognizing palliative care needs. According to Waller et al. the NAT-PD:HF could be useful for routinely assessment. There was no consensus about suitability of the outpatient clinic.

**Table 4.** Context of using the tools in practice.

Tool	Author	Patient characteristics	Professional characteristics	Type of healthcare organization	Format	Timing and setting
IPOS	Kane et al.^28,29^	Patients attending nurse-led CHF clinics; (NYHA class III–IV, with HFrEF, or HF symptoms and either HFmrEF or HFpEF; fluency and literacy in English; sufficient cognitive function to complete the questionnaires; ⩾18 years of age.	Nurses specialized in heart failure	Two national tertiary referral centers	Paper format, on a clip board with a pen	Patients completed the IPOS while waiting to be reviewed at the nurse-led CHF disease management clinic, on arrival to the clinic
NAT-PD:HF	Waller et al.^31^	(1) HF of either systolic or diastolic etiology being managed by a MHFC, (2) receiving optimal therapy or documented intolerance, (3) hospitalized within the last 12 months for CHF, (4) understanding English to complete the questionnaires, and (5) emotionally and cognitively capable of participating.	Clinic staff members	Metropolitan referral hospital	One page tool assessment	In outpatient consultation or during admission (cardiology ward). A second staff member completed a second copy of the NAT: PD-HF during a second consultation for the same participant on the same day without discussing the patient or comparing responses with the ﬁrst staff member
NAT-PD:HF	Janssen et al.^32^	CHF- outpatients, and if present their primary family caregiver. Patients had a diagnosis of CHF NYHA III or IV according to the ESC guidelines; scheduled to receive a home visit by a HFN; able to provide IC, complete written questionnaires or participate in interviews. Patients already known to the specialist PC team were ineligible.	Nurses specialized in heart failure	One university hospital	One page tool assessment	Usual care, home visit
RADPAC	Thoonsen et al.^34–38^	Patients with CHF, COPD or cancer who potentially could benefit from a palliative care approach	General practitioners	General practice	Set of indicators	To screen the medical records of all persons in their practice. General practitioners were also asked to use this screening instrument whenever new data of any patient with one of these three diseases became available
HFNAQ	Davidson et al.^39^	Patients with HF discharged from a hospital within 30 days. NYHA class I to IV, and willing to give IC and attend an educational and exercise program. Those with insufficient language skills and cognitive impairment were excluded from the study.	Cardiac clinical nurse specialist	A tertiary referral center with comprehensive cardiac surgery and inter-ventional cardiology services	Questionnaire	During an assessment to participate in a CHF-specific educational and exercise program Patients had the opportunity to complete HFNAQ with the nurse or take it home and return it within 7 days.
NECPAL	Gastelurrutia et al.^46^	All consecutive ambulatory patients attended in the CHF clinics of 3 university hospitals during a 4-month period were enrolled	Doctors and nursing staff	Three university hospitals	Questionnaire	During a scheduled ambulatory visit
NECPAL	Orzechowski et al.^45^	Patients > 35 years, hospitalized with a HF diagnosis NYHA III/IV or EF ⩽40% in the last 12 months. Exclusion criteria: inadequate cognitive conditions to respond adequately to questions, and absence of a responsible person capable of responding on their behalf.	Physicians	One tertiary hospital	Questionnaire, paper format	A cardiology ward.The researcher asked the NECPAL questions to the physician during routine care

NYHA: New York Heart Association (class I-IV); IC: Informed Consent; CHF: Chronic Heart Failure; HFrEF: Heart Failure with reduced Ejection Fraction (EF<40%); HFmrEF: Heart Failure with midrange systolic function (EF 41-49%; HFpEF: Heart Failure with preserved systolic function (EF >50%); ESC: European Society of Cardiology; HFN: Heart Failure Nurse; EF: Ejection Fraction; MHFC: Multidisciplinary Heart Failure Clinic.

#### Implementation process

No study sufficiently addressed implementation aspects of the tools and the level of implementation in the context of advanced chronic heart failure. Three studies described the tool as a single-faceted implementation strategy.^39,45,46^ No studies described a final multidisciplinary review about the items and a multidisciplinary commitment to use the tool. Neither were frequent evaluation or review processes of the tool’s content described, nor the approval from appropriate stakeholders prior to implementation in the clinical environment.

Education and instruction activities about how to use the tools were described in several studies. Education was developed to train heart failure nurses about how to use the IPOS and the NAT: PD-HF as part of feasibility studies.^28,29,32^ Heart failure nurses attended an educational meeting^28,29,32^ and/or received educational material^28,29^ before they tested the tools in practice. The IPOS was perceived by patients as an easy to fill in PROM without need for additional information and heart failure nurses were instructed not to give their own explanations to questions.^28,29^ The NAT-PD: HF was developed so that health care professionals could complete the tool without training.^31^ According to a pilot testing of the NAT:PD-HF, Waller et al. found that the tool may be more appropriate for healthcare professionals with specific chronic heart failure knowledge.^31^ Janssen et al. showed that the heart failure nurses who piloted the NAT-PD:HF needed more guidance to address the recognized palliative care needs.^32^

Thoonsen et al. reported a strategy used to implement early identification of proactive palliative care planning of palliative patients by the GP.^37^ Early identification was based on two tools: the RADPAC in combination with the Proactive Palliative Care Planning Card. Educational meetings (5 h training), educational materials, educational outreach visits with a physician specialized in palliative care and reminders were used to facilitate early identification of palliative care needs. The education was not developed primarily for advanced chronic heart failure. Despite the RADPAC indicators specifically for advanced chronic heart failure, the actual identification of patients with advanced chronic heart failure and palliative care needs remained challenging. After 1 year of implementation, the intervention group (*n* = 57) and control group (*n* = 77) GP’s identified no patient with advanced chronic heart failure and palliative care needs. Six GPs of the untrained control group (who responded to the control group questionnaire and to the RADPAC intervention questionnaire 3 weeks later) identified four patients with advanced chronic heart failure and palliative care needs after having received the RADPAC. The authors suggested that RADPAC used by GPs, has a positive short-term effect on the awareness of needs in patients with advanced chronic heart failure among GPs.

According to Thoonsen et al. the spiritual dimension of the proactive care planning tool (RADPAC) was often left out or very densely described.^34,35^ Kane et al. showed that the heart failure nurses perceived the spiritual question of the IPOS as very challenging.^29^ The nurses were uncertain about the meaning of this question and were uncertain in what the patient may need. Waller et al. described that the spiritual/existential item of the NAT-PD: HF was potentially difficult to assess.^31^

## Discussion

We identified seven tools that have been developed to help healthcare professionals recognize palliative care needs of patients with advanced chronic heart failure. The tools varied in purpose, items, intended user, and integrated guidance about how to use the tool. The validation of the tools specifically for the context of advanced chronic heart failure and palliative care is limited. Four of the seven identified tools showed some level of validation. The validation was merely based on content validity. The IPOS showed to be the most developed patient reported outcome measurement tool and the NAT-PD:HF showed to be the most validated tool used by health care professionals. No publications were identified focusing on routine use of the tools in daily heart failure practice.

### What this study adds

#### Aim of the tools

The current review reveals that the tools varied in purpose for identification of palliative care needs. This is in line with the findings related to palliative needs assessment tools in Parkinson’s disease.^47^ Richfield and Johnson suggested that different assessments at different points in the care process, with different purposes (e.g. identification, detailed assessment) are needed for a holistic assessment of palliative care needs in Parkinson’s disease. They also identified tools such as the NAT:PD and the IPOS/POS and described that these tools may supplement each other. According to Richfield and Johnson the IPOS/POS could be used to prioritize the possible current patient’s needs before or at the beginning of the consultation. The NAT:PD could be used to check for and direct the recognized palliative care needs. However, combining tools may influence the complexity of a quality improvement intervention and may limit successful uptake of the tools in practice.

#### Content of the tools

Palliative care needs of patients with advanced chronic heart failure are difficult to recognize. Patients with advanced chronic heart failure and their family caregivers have specific palliative care needs that are often neglected in comparison with cancer patients, such as support for the feeling of abandonment and access to medical care, knowledge and understanding about the disease, prognosis, and care.^48,49^ Moreover, patients with advanced chronic heart failure are less likely to report on their symptoms compared to patients with cancer.^50^ What makes recognition also difficult is that the actual perceived patient’s palliative care needs and wishes may vary irrespective of the individual and unpredictable disease trajectory and personal circumstances.^51^ This implies that the tool needs to be broad *and* specific for advanced chronic heart failure to identify the possible needs of the patient (and caregiver). Therefore, not all identified tools may be effective to recognize palliative care needs in the context of advanced chronic heart failure, based on their limited items and the specificity for advanced chronic heart failure. The NECPAL includes general and chronic heart failure specific indicators which are complemented with the Surprise Question. This integration of the Surprise Question may be a barrier for timely recognition of palliative care needs in chronic heart failure as it depends on predicting survival which is difficult in chronic heart failure. This review shows that items regarding chronic heart failure specific medication and treatment regimens may facilitate the health care professional in directing palliative care needs. Furthermore, integrating family caregiver needs,^52^ using a comprehensive list of prompts or symptoms, and leaving room in questions for unique responses may help to identify palliative care needs. However, such unique responses may need more skills, heart failure experience or guidance to interpret the information the patient reveals and communicates.

#### User and interdisciplinary team

Different tools have been developed for different users and were applied in different health care settings. For example, the HeFPAC is a tool which is tailored to the nurses wishes as they desired simplification of information in a tool, whereas the RADPAC includes indicators specifically for general practitioners to prevent to lose track of patients with advanced chronic heart failure. A recent review of Diop et al. showed that an integration of interdisciplinary team interventions regarding advanced chronic heart failure and palliative care is the most promising strategy to improve patient-centered outcomes.^6^ This interdisciplinary work environment may need a general, accessible, and user-friendly tool.

#### Translation into practice

The feasibility and implementability on tool level varied between studies, for example with respect to the NAT-PD:HF.^31,32^ Sufficient time, optimal setting, and heart failure expertise were identified as factors for effective application of the NAT-PD:HF. In the context of interstitial lung disease, the NAT-PD was described as a practical method.^53^ However Reigada et al. also showed that communication training and training to enable holistic assessment facilitate implementation of the NAT-PD in interstitial lung disease care. This indicates that tools may not be enough to improve recognition of palliative care needs due to the local or specific professional needs and that education is needed. Guidance and education for the healthcare professional may have a positive effect on the adaptation and implementation fidelity of using these tools in the context of palliative care.^52,54,55^ The current review shows that educational activities were performed such as educational meetings focusing on how to use the tool and to clarify the concept of palliative care. According to the results, education may also focus on specific chronic heart failure knowledge in relation to palliative care and on how to address palliative care needs. Janssen et al. showed that healthcare professionals need a tool that increases awareness, understanding, and knowledge concerning palliative care needs.^56^ The identified tools do not have clear criterions for referral for palliative care in advanced chronic heart failure. Healthcare professionals may need more structural guidance in when referral is needed. Furthermore, spirituality revealed to be a challenging item to asses by healthcare professionals based on experiences with the IPOS, the NAT:PD-HF, and the RADPAC and therefore needs attention in educational programs.

### Strengths and weaknesses

A strength of this systematic review was the use of different search methods as this makes the search strategy more powerful.^57^ A predefined protocol driven search strategy would not be enough to find the complex and heterogeneous types of evidence we intended to find. Performing the second electronic search including all tools decreased the chance that we missed evidence. Another strength is that we did not exclude references based on language. Validity was assessed based on the performed activities that were described in the research papers. We did not perform a methodological check on the content of these activities, for example on sample size.

Theoretically, it may be possible that conceptual models with multiple patient outcome measurements, such as the Edmonton Symptom Assessment System (ESAS) combined with the Palliative Performance Scale (PPS), would help healthcare professionals with recognition of palliative care needs in patients with advanced chronic heart failure. Nevertheless, they were excluded as they may be too complex for application by healthcare professionals lacking expertise in palliative care.

No studies were identified describing implementation results of tools specifically for advanced chronic heart failure. Though, we are aware that some tools have been integrated in clinical practice guidelines, expert position statements, websites or care programs.^14^ For example, the NECPAL is implemented by the Catalan Department of Health in a general program for the early identification of patients with palliative care needs.^58^

### Implications for future research and practice

The European Association for Palliative Care Task Force expert position statement encourages the use of validated assessment tools to recognize palliative care needs in advanced chronic heart failure.^14^ The Task Force for the diagnosis and treatment of acute and chronic heart failure of the European Society of Cardiology stated that palliative care needs to be timely available for advanced chronic heart failure patients alongside their regular care.^59,60^

Based on our results there are various implications for future research and practice. First, the existing evidence is insufficient and too broad to reflect on which tool may be most promising to facilitate healthcare professionals in recognizing palliative care needs in the context of advanced chronic heart failure. The IPOS showed to be the most validated patient reported outcome measurement and the NAT-PD:HF showed to be the most validated tool that must be completed by health care professionals. More validation research of these and other tools is needed in the context of palliative care and advanced chronic heart failure. Also, the methodology for the development and design of the tools, specifically for advanced chronic heart failure, needs more attention in research. Janssen et al. showed that healthcare professionals in the Netherlands need a tool that is adaptable to different disease stages, facilitates early identification of palliative care needs and eases open conversations about palliative care.^56^ They showed that the complexity of chronic heart failure should be considered in a personalized approach. Depending on the target user and the aim of a future tool, researchers should consider using the IPOS and the NAT-PD:HF as a starting point in the refinement and validation of an existing tool or the development of an new tool.

Second, to increase the chance for uptake of a tool in for example heart failure clinics or primary care, there must be a balance between simplicity of a tool, and instruction and education. The needs for instructions and education may vary between disciplines due to the setting and the level of expertise. Other factors that need to be taken into account are optimal setting and time to complete the tool. More studies are needed to explore the desired intervention characteristics of the end users and to explore how these tools can be integrated into practice.

Third, it is unknown to what extent a tool is needed specifically for advanced chronic heart failure. For example, the IPOS is not a disease specific PROM, but seems to be promising to identify palliative care needs in patients with advanced chronic heart failure. Therefore, researchers must explore to what level tools differ with respect to advanced chronic heart failure and other chronic life-limiting conditions such as advanced chronic obstructive pulmonary disease.

## Conclusion

There are tools available that are only validated to a limited extent to recognize palliative care needs in patients with advanced chronic heart failure. The IPOS and the NAT-PD:HF are the most validated tools in the context of palliative care and advanced chronic heart failure. This review concludes that there is a need for a validated and a more feasible tool to facilitate healthcare professionals in recognizing these needs. Guidance and education may facilitate the uptake and correct application of these tools in practice.

## Supplemental Material

Supplementary_File_2_Search_Strategy_Medline – Supplemental material for Tools to help healthcare professionals recognize palliative care needs in patients with advanced heart failure: A systematic review Click here for additional data file. Supplemental material, Supplementary_File_2_Search_Strategy_Medline for Tools to help healthcare professionals recognize palliative care needs in patients with advanced heart failure: A systematic review by Stephanie MC Ament, Inge ME Couwenberg, Josiane JJ Boyne, Jos Kleijnen, Henri EJH Stoffers, Marieke HJ van den Beuken, Yvonne Engels, Louise Bellersen and Daisy JA Janssen in Palliative Medicine

Supplementary_File_3_Electronic_Search_Strategy_Methodological_check_Medline – Supplemental material for Tools to help healthcare professionals recognize palliative care needs in patients with advanced heart failure: A systematic review Click here for additional data file. Supplemental material, Supplementary_File_3_Electronic_Search_Strategy_Methodological_check_Medline for Tools to help healthcare professionals recognize palliative care needs in patients with advanced heart failure: A systematic review by Stephanie MC Ament, Inge ME Couwenberg, Josiane JJ Boyne, Jos Kleijnen, Henri EJH Stoffers, Marieke HJ van den Beuken, Yvonne Engels, Louise Bellersen and Daisy JA Janssen in Palliative Medicine
