# Supplementary material for: Tools to help healthcare professionals recognize palliative care needs in patients with advanced heart failure: A systematic review
Source: Palliat Med. 2020 Oct 15;35(1):45–58. doi: 10.1177/0269216320963941 (PMC7797617; doi:10.1177/0269216320963941)
Supplement: Supplementary_File_2_Search_Strategy_Medline – Supplemental material for Tools to help healthcare professionals recognize palliative care needs in patients with advanced heart failure: A systematic review [file Supplementary_File_2_Search_Strategy_Medline.docx]

**Supplementary file 2: Electronic search strategy to identify tools via Medline**

Database: Ovid Medline <1946 to July 10, 2019>

Date searched: July 11, 2019

Records found: 399

1. Tool:
   1. (identif$ adj4 (tool or instrument or directive or checklist or asses$ or scale or questio$ or method or model or guidance or evaluat$ or monitor$)).ti,ab,ot.
   2. (signal$ adj4 (tool or instrument or directive or checklist or asses$ or scale or questio$ or method or model or guidance or evaluat$ or monitor$)).ti,ab,ot.
   3. Needs Assessments/
   4. "Surveys and Questionnaires"/
2. Palliative care:
   1. (end adj4 (life or stage)).ti,ab,ot.
   2. (advanced adj4 (stag$)).ti,ab,ot.
   3. palliat$ or EOL or terminal$ or supportive or incura$ or dying.ti,ab,ot.
   4. symptom adj4 (manage$ or control or burden).ti,ab,ot.
   5. suffer$ or (patient adj4 needs) or (life adj4 threat$).ti,ab,ot.
   6. Terminally ill/
   7. Palliative care/
   8. Hospice care/
3. CHF
4. (Chronic heart) adj4 failure.ti,ab,ot.
5. (congestive heart) adj4 failure.ti,ab,ot.
6. Advance$ adj2 heart.ti,ab,ot.
7. CHF. ti,ab,ot.
8. Heart failure/
9. 1 AND 2 AND 3: 405
10. exp animals/ not (exp animals/ and humans/): 6
11. 4 NOT 5: 399
