# Supplementary material for: Tools to help healthcare professionals recognize palliative care needs in patients with advanced heart failure: A systematic review
Source: Palliat Med. 2020 Oct 15;35(1):45–58. doi: 10.1177/0269216320963941 (PMC7797617; doi:10.1177/0269216320963941)
Supplement: Supplementary_File_3_Electronic_Search_Strategy_Methodological_check_Medline – Supplemental material for Tools to help healthcare professionals recognize palliative care needs in patients with advanced heart failure: A systematic review [file Supplementary_File_3_Electronic_Search_Strategy_Methodological_check_Medline.docx]

**Supplementary file 3: Electronic search strategy to check for evidence via Medline**

Database: Ovid Medline <1946 to June 2019>

Date searched: 13.09.2019

Records found: 🡪 380

1. Tool:
   1. ((“Integrated Palliative care Outcome Scale”) or IPOS or (“Palliative care Outcome Scale”) or POS or (“Needs Assessment Tools Progressive Disease”) or (“NAT: PD-HF”) or (“Supportive and Palliative Care Indicators Tool”) or SPICT or (“RADboud indicators for PAlliative Care Needs”) or RADPAC or (“Heart Failure Needs Assessment Questionnaire”) or HFNAQ or (“Care related Quality of Life for Chronic Heart Failure Questionnaire”) or CareQol CHF or (“Heart Failure Palliative Approach to Care”) or HeFPAC or (“Necesidades Paliativas”) or NECPAL).ti,ab,ot.
2. Implementation: a or b
   1. (Adopt* or diffuse* or disseminat* or institutionali?ation or implement* or routini* or normali?ation or (knowledge translation) or uptake or applicab* or compatib* or mainten* or durability or (integration of practice) or sustain* or penetrat*).ti,ab,ot.
   2. *implementation science/
3. 1 AND 2
4. humans/
5. 3 AND 4
